# Mercurius-vivus

**Published:** 1896-09

**Authors:** 


### HOMŒOPATHIC PHYSICIAN,

A MONTHLY JOURNAL OF

HOMŒOPATHIC MATERIA MEDICA AND CLINICAL MEDICINE.

"If our school ever gives up the strict inductive method of Hahnemann, we are lost, and deserve only to be mentioned as a caricature in the history of medicine."—CONSTANTINE HERING.

Vol. XVI. SEPTEMBER, 1896. No. 9.

### EDITORIAL.

MERCURIUS-VIVUS.—Dr. Guernsey's key-notes of Mercurius are as follow: Much salivation; a constant driveling from the mouth. Convulsions mostly in the extremities. Much perspiration, affording no relief. Constant short, choking cough. Anguish. Prolapsed vagina. Lancinating, boring, or pressing pains. Moist tongue with intense thirst. Always worse throughout the night.

Leucorrhœa worse at night. Leucorrhœa causing itching, burning, smarting, corroding, or sensation of rawness. Sometimes discharges from the vagina of pus and mucus in flocks as large as hazel nuts.

During menstrual period anxiety, red tongue with dark spots on it and burning, salty taste in the mouth, sickly color of the gums, and teeth set on edge. Cold and clammy sweat on thighs every night. Symptoms worse in cold, damp weather. Moist skin; soreness of the throat; soreness of the inguinal glands. Indurated tumor of the vagina with raw, sore feeling. Inflammatory swelling of the internal surface of the vagina. Long-lasting itching of vulva shortly before the menses. The itching is aggravated by even a single drop of urine. It must be immediately washed off. Pimples or tubercles on the labia, more troublesome at night. Symptoms always worse in bed. Sensation of coldness in the ears, continually. Morning diarrhœa mostly of slime, before and during stool. Diarrhœa preceded by faint "sickish" pain in the abdomen entirely relieved by stool.

Dr. Bell, in his book on Diarrhœa, gives as the great characteristic of Mercurius, tenesmus and urging before, during, and after stool. "A sort of never-get-done feeling."

